# Diagnosis of gestational diabetes in Uganda: The reactions of women, family members and health workers

**DOI:** 10.1177/17455065211013769

**Published:** 2021-04-30

**Authors:** Flavia Zalwango, Janet Seeley, Arthur Namara, Sanjay Kinra, Moffat Nyirenda, Laura Oakley

**Affiliations:** 1 MRC/UVRI and LSHTM Uganda Research Unit, Entebbe, Uganda; 2 London School of Hygiene and Tropical Medicine, London, UK; 3 Centre for Fertility and Health, Norwegian Institute of Public Health, Oslo, Norway

**Keywords:** diabetes, East Africa, healthcare, pregnancy, women’s health

## Abstract

**Objectives:**

In Uganda, as in many other low- and middle-income countries, screening for gestational diabetes mellitus is suboptimal and is rarely embedded in routine antenatal care. We describe the experiences of women in Uganda who underwent screening for gestational diabetes mellitus and were diagnosed with the condition as they navigate both the reaction of family members and their interaction with health workers.

**Methods:**

Pregnant women aged 18 years or older and between 24 and 28 weeks of gestation were enrolled from the antenatal clinics at one of the five hospitals between 13 June 2018 and 31 October 2019. Ten women with gestational diabetes mellitus, ten family members and six health workers were purposively selected to take part. Interviews and focus group discussions were used to collect data on the socio-cultural and health system factors that influence timely screening and effective management of gestational diabetes mellitus in Uganda. Data were analysed thematically.

**Results:**

Women generally reflected on the importance of gestational diabetes mellitus screening and felt that an early diagnosis helped them to get timely medical attention, and most reported a positive experience of the care provided by health workers. However, women who were diagnosed with gestational diabetes mellitus reported feeling fearful and anxious, and some were worried that the condition might be life-threatening. Many women reported that they were upset and largely unprepared to receive a gestational diabetes mellitus diagnosis. A gestational diabetes mellitus diagnosis not only stirred intense feelings of fear and anxiety in women but also affected their spouses and other family members. Many male partners were sympathetic and willing to provide support.

**Conclusion:**

Our findings highlight the need to understand the perceptions and emotions that accompany a gestational diabetes mellitus diagnosis to best support women and their family members. An improved recognition of these factors can inform the development of effective gestational diabetes mellitus screening and management programmes.

## Background

Gestational diabetes mellitus (GDM), the most common metabolic disorder of pregnancy,^1^ is defined as a type of glucose intolerance diagnosed in the second or third trimester.^2^ Women with GDM are at increased risk for adverse outcomes like foetal macrosomia, obstructed labour, birth injuries, and maternal and perinatal mortality.^3^ Although women with GDM revert to normal glucose metabolism after delivery, they are at risk of developing type 2 diabetes later in life, as are their offspring. Early detection and optimal management of the condition is vital to ensure better maternal and foetal outcomes. In Uganda, as in many other low- and middle-income countries (LMICs), screening for GDM is suboptimal and is rarely embedded in routine antenatal care. A recent systematic review estimated a GDM prevalence of 9% across sub-Saharan Africa,^4^ although the authors reported significant heterogeneity in estimates due to a lack of organized screening programmes in this region.

A number of studies have focussed on the experiences of women diagnosed with GDM,^5–9^ but a few have investigated women’s reactions following diagnosis.^10,11^ Craig et al.^11^ reviewed 41 studies (two were conducted in LMICs and none were from Africa) to understand the psycho-social experiences of women following a GDM diagnosis. Their findings showed that as part of the initial impact women felt ‘self-blame, failure, fear, sadness, concern and confusion’ (p. 4). The authors highlight the importance of psychological and social support to help women manage their diagnosis. A study in Tehran, Iran, conducted in 2016 showed that the fear of receiving a diabetes diagnosis at the follow-up visit greatly affected post-partum follow-up care in women with recent GDM.^12^ In a study among migrant African women in Sweden with GDM,^13^ women were found to be concerned about having to adjust their way of life because of their diagnosis, the risk of developing type 2 diabetes and fear of being on long-term treatment.

While Craig et al.^11^ reported negative feelings and the need for support, they also found that some women saw the diagnosis as an avenue for self-improvement or a ‘wake-up call’. Similarly, a study^14^ conducted in Australia showed that women were determined to improve their lifestyle following the diagnosis.^10^

Little is known about women’s reactions to a GDM diagnosis in the sub-Saharan setting. The ‘lived’ experience of being diagnosed with GDM will be shaped by many contextual factors, including the ability and preparedness of health care services to provide support and appropriate follow-up to women with GDM, for instance, by employing both internationally and locally supported clinical guidelines and equipping them with relevant and clear information,^15^ social and family support networks, and wider understanding and recognition of the condition.

In this article, we describe the experiences of women in Uganda who underwent screening for GDM and were subsequently diagnosed with the condition. In addition, we describe the experiences of family members and health workers.

## Methods

### The study setting

The data for this article were drawn from a sub-study of a larger work programme focusing on gestational diabetes in Uganda, conducted by the Medical Research Council/Uganda Virus Research Institute and London School of Hygiene and Tropical Medicine (MRC/UVRI and LSHTM) Uganda Research Unit. Study participants, pregnant women aged 18 years or older and between 24 and 28 weeks of gestation, were enrolled from the antenatal clinics at one of the five hospitals between 13 June 2018 and 31 October 2019.

This work is based around two separate studies: a cross-sectional study rolling-out GDM screening in participating clinics and a stand-alone randomized controlled trial evaluating an educational intervention trial designed to improve the screening and management of GDM in 30 antenatal clinics in Wakiso and Mpigi Districts (NIH registry (www.clinicaltrials.gov), registration no. NCT03937050 (registered 3 May 2019)). The former study was nearing completion at the time the data reported here were collected, and the facilities and participants involved in this screening study formed the study population for the sub-study.

The screening study which provided the setting for this sub-study was conducted at two public hospitals in Wakiso and Masaka Districts in Central Uganda – Entebbe Regional Referral Hospital (urban) and Masaka Regional Referral Hospital (rural). As part of this study, women attending for antenatal care were screened for GDM using an Oral Glucose Tolerance Test (OGTT) between 24 and 28 weeks of gestation. Before GDM screening, women were given health education and reassured that if they tested positive for GDM, their condition could be treated. Screening took place on weekday mornings during routine antenatal clinics. Blood samples were taken after an overnight fast, and screened for HIV, syphilis and anaemia in addition to GDM. The nurse/midwife also measured the mothers’ weight, height, blood pressure, and waist and hip circumference. Alongside the screening programme, health workers conducted health education sessions for women. During these health education sessions, health workers explained that GDM was a pregnancy-related condition, usually diagnosed between 24 and 28 weeks of gestation, and that one could still have a good pregnancy outcome following a GDM diagnosis. Women were informed of screening results within 3 weeks, and those diagnosed with GDM were referred for specialist care.

## Methods

### The sample

Participants in this sub-study were purposively selected from those who had been involved in the GDM screening study at Entebbe Regional Referral Hospital and Masaka Regional Referral Hospital. Participants included pregnant or post-natal women who had undergone GDM screening, and had a GDM diagnosis, family members and health workers.

### Data collection

We used semi-structured interviews, Key Informant Interviews and Focus Group Discussions to collect data on the socio-cultural and health system factors that influence timely screening and effective management of GDM in Uganda. We conducted fieldwork between 17 June and 3 July 2019. Data were collected from 10 pregnant or post-natal women with GDM, 10 family members and 6 health workers (four nurses and two doctors with experience in managing GDM). We also conducted two Focus Group Discussions with nine women with GDM in Masaka. Interviews were conducted in Luganda or English and were audio-recorded. These were later transcribed, and interviews in Luganda were translated into English.

### Data management and analysis

Data were analysed thematically and managed using Nvivo 12. We developed a coding frame based on the study objectives and these were augmented with new themes emerging from the data. Data were coded under the various thematic categories and thematic summaries were written on each of the main themes. Each participant was assigned a pseudonym and these are used in this article. Data were analysed using descriptive and interpretive phenomenology approaches. A social realist theoretical framework was used to reveal individual experiences of a GDM diagnosis.

The research project was approved by the research and ethics committee of Uganda Virus Research Institute (approval no. GC/127/19/04/625) and Uganda National Council for Science and Technology (approval no. HS2340). The women, health workers and family members gave written informed consent to participate in the study and the use of the material from the interviews, including quotes, in this article.

## Results

In total, 10 pregnant or post-natal women aged 18–40 years, who had been diagnosed with GDM, were interviewed (Table 1). We also conducted one Focus Group Discussion with nine women with GDM in Masaka. Most of the women had some secondary education, and the majority were not in paid employment. For those who were employed, the main occupations included petty trade, hairdressing and tailoring, and a few were in formal employment. Six of the women said they had a family history of type 2 diabetes.

**Table 1.** Participant socio-demographic characteristics.

Age (years)	GDM status	Number of children	Pregnancy trimester
18	GDM	0	3
23	GDM	1	3
26	GDM	2	3
28	GDM	3	3
29	GDM	2	6
30	GDM	3	Post-natal
30	GDM	4	Post-natal
30	GDM	2	Post-natal
34	GDM	0	Post-natal
40	GDM	3	Post-natal

GDM: gestational diabetes mellitus.

The family members interviewed included one mother-in-law, four mothers and seven sisters, with an overall age range of 25–69 years. Eight spouses (aged between 22 and 43 years) were also interviewed. Half of the family members interviewed had attained primary education and one man and two women had attained post-secondary education.

The nine participants in the Focus Group Discussion for women with GDM were aged between 21 and 39 years (six were aged below 27 years). One had tertiary education, three had primary education and four had secondary education. Four were petty traders, one was a farmer and three were not working outside the home. One woman earned less than 200,000 shillings per month, five earned 200,000–300,000 shillings (£40–£60) and three earned 350,000–500,000 shillings (£70–£100). The health workers included two medical doctors (gynaecologists) and four registered nurses/midwives.

### General family situation of the pregnant women

Before describing the findings related to GDM, we describe briefly the family influences and circumstances of the women to provide the context for their experience of diagnosis. Although financial problems were commonly reported by the women, some suffered other difficulties which affected their access to care and support during pregnancy.

Most of the women reported receiving support from their husbands and other family members (sisters, mothers and mothers-in-law) throughout their pregnancy. Husbands usually provided financial support, paid for hospital bills and other necessities while some escorted their wives to the hospital to access antenatal care services. Husbands also played a vital role in supporting pregnant mothers who owned businesses either by attending to these businesses or by monitoring them in their absence. Some women reported that female relatives helped with domestic chores like cooking, washing, fetching water, cleaning the home, taking care of domestic animals, baby-sitting existing children and escorting them to the hospital for antenatal care. In addition, several parents-in-law provided financial support to the women.

However, some of the interviewees reported that they did not receive any financial or emotional support from their husbands or family members during their pregnancy. Women attributed this to various reasons, for example, alcoholism and domestic violence (e.g. when a woman did not wish to have sexual intercourse with her husband). In such cases, women had to find alternative forms of securing support. We found that some women were proactive in seeking an income through providing casual labour on other people’s farms, washing peoples’ clothes, while others started small-scale businesses like selling potato fries, roasted maize and porridge by the roadside.

### Processes and roles in GDM screening

#### Women’s reactions immediately following diagnosis

Women generally reflected on the importance of GDM screening and felt that an early diagnosis helped them to get timely medical attention, and most reported a positive experience of the care provided by health workers.

However, following diagnosis, women who tested positive for GDM reported feeling emotional and fearful, and some harboured thoughts about death. With limited GDM screening undertaken outside the study context, women rarely reported knowing of others with the same diagnosis. A few said that they did not have any negative feelings, perhaps because they did not believe the diagnosis.

When one man was asked about his wife’s reaction after GDM diagnosis, he explained that she had been shocked and worried by the news. This was evident from his report of phone calls she had made to different people, including her mother, when she received her diagnosis. ‘They cannot tell you that you have such an illness and you fail to get worried’, he said. He also noticed a change in her behaviour and said that they both worried about their children. Similarly, another 40-year-old woman said that after the diagnosis, she called her mother to inquire whether their family had other people with diabetes. She calmed down after the mother explained to her that the disease was probably caused by pregnancy.

Many women like Allen (26-year-old woman) also reported that they feared that they would die and leave their children without any one to support them:

I was scared and shocked; I saw as if my life had ended and that I was not going to be healthy and my baby would live with diabetes. I thought about [leaving my young children behind].

Where the women had known or heard about a few individuals who previously suffered from diabetes, they imagined that they would go through the same suffering or incur a heavy financial burden to treat the disease. Women feared that they would develop sores or have to inject themselves every day, eat special foods or take pills ‘like a daily meal’:

In fact it disorganized me because if I hear from people who live with that sickness, they say that it is not good. It can kill you anytime. [I fear] that I may die and leave my children to suffer. (Sumaya, 30-year-old woman)

Women reported wondering about the cause of their condition, and expressed anxiety at the prospect of taking daily injections or even giving birth to a child with abnormalities. Some women were not sure whether GDM was treatable or curable, and felt that little information had been given regarding the implications of a GDM diagnosis. When asked about what GDM-related changes the doctor had talked to her about, Allen, a 26-year-old woman said:

He didn’t tell me anything and he didn’t have time to talk to me since he had many patients to attend to. After testing you, he tells you about your GDM status and gives you the review date.

Many women reported that they were upset and largely unprepared to receive a diagnosis of GDM. Some struggled initially to come to terms with their diagnosis and what it meant for themselves, their family members and the pregnancy. Some women were unsure of how to disclose their diagnosis to their partner. Those who did disclose said that the process elicited feelings of sadness.

Some participants immediately thought about death and what would become of their young children. Others thought about where to get the finances to manage GDM.

Notably, some participants received reassurance from health workers that GDM is a short-lived illness, and these women therefore reported that they did not consider GDM a serious disease.

Health workers, however, said that anxiety was a common response following a GDM diagnosis. Many women were taken by surprise and had questions about what they could do to ensure the health of their baby:

I don’t know anything because they have just disclosed to me and it is new to me. So I just need advice from you on what to do and what not to do. Now I don’t know. I don’t know what to eat and what not to eat. It is important they advise first and we see how we can [follow] the doctor’s advice. (Rita, 28-year-old woman)

Since I have just been diagnosed with GDM and we don’t have any family member with GDM, I know nothing about it. I don’t understand anything about GDM. (Brenda, 26-year-old woman)

The health workers said that diabetes was one of the ‘most feared diseases’ in the antenatal setting and that women who came to the hospital were desperate to know what would happen next after GDM diagnosis. They usually counselled the women in order to allay their fears. The health workers said that they reassured the women that the health workers would take care of them at minimum cost and that the condition resolves after delivery:

Since it is their first time to get such news some of them come when they are worried but after counselling they relax. [We reassure] them that it is not the end. (Nurse, Entebbe)

Usually these women are too scared when they receive a positive diagnosis, they think it is the end of the [world]; they think the diabetes will not be cured or it will be passed on to their babies. (Nurse, Masaka)

Following a GDM diagnosis, the health care providers reported that it was at the women’s discretion to disclose or not to disclose their diagnosis to their husbands; most women did not.

The health workers further observed that some women were anxious about their longer-term health. Health workers were able to explain to women with GDM that it is a temporary condition. They reported that many women were reassured by the prospect of a postnatal OGTT to confirm that their glucose intolerance had resolved.

### Reactions of family members immediately following diagnosis

GDM diagnosis not only stirred intense feelings of fear and anxiety in women but also affected their spouses and other family members in various ways. Husbands who were told about the diagnosis thought about their wives dying or were concerned about the financial costs of treatment:

On the first day she disclosed to me, I told her these are problems! Where am I going to get money for treatment? The second one is that, if my wife dies now, how am I going to bring up all these kids alone? Stepmothers are there but they will not treat my kids the way their mother treats them. (Issa, 36-year-old man)

When asked about her husband’s reaction towards her GDM diagnosis, one woman said that he started blaming her for taking too much sugar. Another woman said that after she shared about her diagnosis with her sister, she told her it must be true ‘because of my weight and social ways’. This was stigmatizing for her. Another woman said that her husband said that she had been given the wrong results and insisted she be tested again at another health centre. He only accepted her diagnosis once it had been confirmed again.

In other narratives, family members were sympathetic and willing to help the women access and adhere to their treatment schedules. Some family members said that they felt strong once they were reassured by health workers that the condition would disappear after the women gave birth:

She [mother] seemed scared and said that we are suffering from diseases which she has never suffered from but I told her that I was told that the disease would be cured. She relaxed after I told her this. (Amina, 30-year-old woman)

Although a few men blamed their partners for not taking good care of themselves, many were sympathetic and willing to help them through their ordeal.

### Challenges in GDM screening and diagnosis

The health workers reported that follow-up after GDM screening was challenging because eligible mothers did not deliver their babies from the hospitals where they were screened. As a result, cord blood samples, for testing, could not be drawn. In addition, many mothers began antenatal care clinic attendance very late (at 36–38 weeks) and this made management of their condition difficult. In addition, some health workers reported delays in the screening process due to stock outs of testing kits and other supplies needed for the management of GDM.

## Discussion

The findings from this study suggest that women who were diagnosed with GDM reported feeling fearful and anxious, and some were worried that the condition might be life-threatening. Many women reported that they were upset and largely unprepared to receive a GDM diagnosis. Some struggled initially to come to terms with their diagnosis and what it meant for their pregnancy and immediate family. This is consistent with results reported by previous studies.^5,12,13,16^ Fear and anxiety post-diagnosis are probably partly driven by a lack of understanding regarding the implications of a GDM diagnosis. In settings where GDM screening is routine, healthcare workers will be more knowledgeable and therefore better equipped to help women navigate a GDM diagnosis. Similarly, in such settings, pregnant women are more likely to know of others with the same diagnosis and can be reassured that GDM is a temporary condition and that treatment will minimize the risks to themselves and their baby.

Our findings also suggest that some women felt reassured that GDM was treatable, and they were keen to take action to minimize the impact on their health and that of their baby. This is consistent with the finding by Craig and colleagues^11^ that some women looked at a positive result as an avenue for self-improvement and also the finding from the study by Han et al.^10^ where more than half of the women said that they were neither concerned nor worried about the diagnosis.

The perceptions of family members after a GDM diagnosis are largely absent in existing literature. Family and social support are key facilitators for health seeking and also treatment adherence.^15^ Family members who were interviewed in this study reported a range of concerns including the financial costs associated with diagnosis.

### Implications

This study has demonstrated that GDM screening programmes need to consider the context in which women receive and process a GDM diagnosis. In settings with little GDM screening, GDM awareness and knowledge are often poor, even among health workers,^17^ highlighting a need to raise awareness about the condition.

### Study limitations

While half of the women included in this study were pregnant and had been diagnosed with GDM, we also included five women who had suffered from GDM but had already delivered their babies. This was because the number of women diagnosed with GDM and still pregnant was few. The women who had already delivered, therefore, recounted their reactions to GDM diagnosis retrospectively, which may have affected their responses. The focus group discussions lasted for 4 h because the women were eager to share their experiences; however, while the women took breaks during this time, the length of the meeting may have affected the engagement, as they were tired.

## Conclusion

In this article, we described the experience of women after GDM diagnosis. For most women, a diagnosis of GDM was associated with fear and anxiety. Our findings highlight the need to understand the perceptions and emotions that accompany a GDM diagnosis to support women and their family members better. An improved recognition of these factors can inform the development of effective GDM screening and management programmes.

## Supplemental Material

sj-pdf-1-whe-10.1177_17455065211013769 – Supplemental material for Diagnosis of gestational diabetes in Uganda: The reactions of women, family members and health workers Click here for additional data file. Supplemental material, sj-pdf-1-whe-10.1177_17455065211013769 for Diagnosis of gestational diabetes in Uganda: The reactions of women, family members and health workers by Flavia Zalwango, Janet Seeley, Arthur Namara, Sanjay Kinra, Moffat Nyirenda and Laura Oakley in Women’s Health
