# Supplementary material for: Diagnosis of gestational diabetes in Uganda: The reactions of women, family members and health workers
Source: Womens Health (Lond). 2021 Apr 30;17:17455065211013769. doi: 10.1177/17455065211013769 (PMC8111520; doi:10.1177/17455065211013769)
Supplement: sj-pdf-1-whe-10.1177_17455065211013769 – Supplemental material for Diagnosis of gestational diabetes in Uganda: The reactions of women, family members and health workers [file sj-pdf-1-whe-10.1177_17455065211013769.pdf]

Supplementary file

GUIDES – STUDY TOOLS

Focus Group Discussion (FGD) Guide: Women with GDM

Group Type: WW GDM / No

Hospital Code:

INTERVIEW INFORMATION:

Interviewer/moderator code	
FGD venue	
Interview date	
NO. OF PARTICIPANTS	
FGD start time	
FGD end time	
Total recorded time (hh/mm/)	

RESPONDENT (R) INFORMATION:

R No	Age	Education (self)	Education (spouse)	Religion	Language (mother	Employment status	No of Children	Pregnancy - ORDER	Pregnancy - TRIMISTER	Diabetes history -	Diabetes history- SELF	Family Type- joint/nuclear	Monthly family
1													
2													
3													
4													
5													
6													
7													
8													
9													

OBSERVATION:

OBSERVATION:

GUIDE:

Pregnancy: Relevance, Knowledge and practices

1. What does it mean to be healthy?
   Kitegeza ki okuba omulamu ?

2. How valuable is women's health in our society?
   Obulamu bw'omukyala bwamuwendo butya mukitundu kyaffe?

a. What influences health behaviour among women?
   Biki ebretera abakyala okulabirira obulamu bwabwe?

b. What factors prevent women from taking care of their health?
   Kiki ekiziyiza omukyala okulabirira obulamu bwe?

3. How important is pregnancy for a woman?
   Olubuto lwa mugaso ki eri omukyala? /olubuto lugasa lutya omukyala?

a. What changes it brings in life:
   Enkyukakyuka olubuto lwezireta mu bulamu

   i. Positive changes due to pregnancy
      Enkyukakyuka enungi mukubeera olubuto

   ii. Difficulties of being pregnant.
      Obuzibu mu kubeera olubuto

4. How women know if a pregnancy is going well (if everything is fine)?
   Abakyala bategeera batya nti olubuto lutambula bulungi (oba buli kimu kiri bulungi)
   a. What are those signs/symptoms that indicate you that you are doing well?
      Bubonero ki obulaga nti oli bulungi?

5. How a pregnant woman should take care of herself?
   Omukyala ali olubuto yerabirira atya?

   a. What are the challenges of doing that?
      Kusomozebwa ki okuli mu kukola ekyo ?

   b. What stops women from taking care of herself?
      Ki ekikomya abakyala okwerabirira

   c. Who else supports women during pregnancy?
      Ani omulala ayamba abakyala nga bali lubuto?

Family: Dynamics, and pregnancy care decisions within the family

6. Generally, who in the family guides/advice women about pregnancy care?
   Ani akuwa amagezi ku by'okulabirira mbuto?

a. How families support women during pregnancy?
   Ab,omumaka bayamba batya abakyala nga Bali mbuto?

b. What do women do in the absence of family support?
   Abakyala bakola ki awatali buyambi bwa famire?

7. Tell me about the decision-making process (with respect to health care seeking) within a family.
   Mbulira ku mitendera gyemuyitamu okusalawo, (nga byekuusa ku kunoonya obujanjabi ewaka?)

a. Who in the family makes decisions (which hospital/doctor, when to visit, etc) regarding health care seeking?
   Ani mu makaago/gamwe asalawo ku by'obulamu (ddwaliro/musawoki, ddi lwe mugenda mudwaliro ne birala)

b. How those decisions are made?
   B'ani abasalawo ?

c. On what basis (criteria- e.g. money, distance, parityetc) decisions are made?
   Okusalawo kuno kukolebwa kutya? (emitendera-nga Sente, obuwanvu, omwenkanokano nebirala)

d. Tell me about women's involvement in decision making with respect to;
   Mbulira okwenyigira kwo mukusalawo ku bino wamanga;

   i. Getting pregnant/ having children
      Ebyo kufuna olubuto / okuzaala abaana

   ii. Choosing a hospital, and the doctor
      Ekusalawo eddwaliro wamu n'omusawo

8. How does a husband support a pregnant woman? Why not (IF NOT)?
   Omwami ayamba atya omukyala ali olubuto? Lwaki nenda (weba nenda)

a. How they should support? What are some of the women's expectations from husbands, particularly during pregnancy?
   Balina kuyaba batya ? bikki kwebyo abakyala bye ba subira mu baami okusingira dala nga bali mbuto?

10. What are those problems (tensions) within your family that affect the pregnancy care? And how? (Probe for Financial, Behavioral- violence, alcoholic members, Social- preference for sons or daughters, Relationships dynamics: In-laws, Husband- wife power, TBAsetc)
   Bizibuki/Mitawaana ki mu maka gamwe ebikosa endabirira y'olubuto? Ate bitya? (Mubyenfuna, Muneyisa, okulwana, abanywi b'omwenge

awaka, Okwagala enyo omwana w'obulenzi, Enkyukakyuka mu mukwano: Ab'enganda, omwami-omuyala nabalala , Obukulembezi)

Culture: pregnancy-related beliefs and practices:

11.What are the beliefs and rituals followed during pregnancy? How they help pregnancy?

Nzikiriza ki n'obulombolombo abantu bwe bagoberera nga bali mbuto? Biyamba bitya?

a. Are these ceremonies/rituals specific to your caste/community?
Gino emikolo/obulombolombo bukozesebwa mu kitundu kyo kyoberamu?

b. What beliefs and practices make the life of pregnant women difficult? How?
Nzikiriza ki awamu n'obulombolombo obukalubiliza obulamu bw'abakyala ab'embuto?

12. Tell us about barriers for a pregnant woman from your caste/ community, to seek health care?
Tubulire ku bukwakulizo ku bakyala ab'embuto mu kitundu kyo ky'obelamu .

13. What are the expected (behaviors) of a pregnant woman? (Probe for Going to hospital, Taking injections/tablets, Doing routine work, Rest /sleep, Food, Cloths)
Omukyala w'olubuto alina kweyisa atya?(Ng'angenda mudwaliro, Ng'afuna empiso/ edagala, Ng'akola emrimu egyabulijjo, Ng'awumudemu/yebase, Mubyendya, Munyambala

14. Please tell about the place where you live (which locality)?
Nsaba ombulire gy'obeera? (kitunduki)

a. What type of population groups (by religion, economic class, education, occupation etc) live there?
Bantu kika ki (mubyenzikiriza, ebyenfuna, mubyenjigiriza, n'emirimu gyebakola) ababeera mukitundu gy'obeera

b. How do neighbours help you? Do women discuss pregnancy related information with your neighbours?
Baliranwa bo bakuyamba batya? Otera okunyumyamu nebalirwanabo ku bikwata ku by'okubera olubuto?

c. How well connected to seek health care? What are benefits living in this area?
Kikwanguyira kitya okufuna obujjanjabi?Birungi ki ebiri mu kubeera mu kitundu kino?

Health Information seeking:

15. Where do women seek information about health care, particularly pregnancy care? From whom?,From where? Or How? (Probe for magazine, internet, social media, WhatsApp/Facebook, TV/Radio etc, Friends/ family/relatives, Neighbours)
Abakyala bafunira wa amawulire agakwata ku by'obujanjabi okusingira dala ku by'okulabirora olubuto? Ani akuwa amawulire ago? Okuva wa? Oba otya? (Empapula/obutabo,

Experience of seeking health (pregnancy) care services:

16. Tells me about your experience of seeking pregnancy care in this hospital (mention the name)?
Mbulira by'oyiseemu mukunywa eddagala nga oli lubuto mudwaliro lino (Yogera erinya ly'eddwaliro).

a. How is the doctor? How does he/she treat you?
Omusawo ali atya? Era akuyisa atya?

b. Does he/she give adequate information/ and reasonable time etc
Akuwa amawulire agasaana n'obudde obumala?

c. How other staffs (nurse/ lab technician) treat you?
Abasawo abalala bakuyisa batya?

17. What do you like about this hospital/health centre?
Kiki kye wenyumirizamu ku dwaliro lino?

18. What do you NOT like about this hospital/health centre?
Kiki kyotayagala ku dwaliro lino?

19. What are suggestions to improve the patient experience at this hospital/health centre?
Biki by'olowooza ebyandikoleddwa ku mutindo gw'obujanjabi mu dwaliro lino?

Knowledge specific to GDM:

Generally, some women are susceptible to a few health complications during pregnancy. GDM is one such condition, where blood sugar level increases. Now, we would like to know more from you, about GDM.
Okutwaliza wamu, abakyala abamu bali mukatyabaga kokufuna obuzibu nga bali mbuto. Obulwadde bwa sukali bwe bumu ku bwo. Twandyagadde okumanya ekisingako okuva gy'oli ebikwata ku sukaali.

20. How many of you know about GDM? Raise hands
Kuffe bameka abamanyi ku sukkali ? tuwanikeko emikono

a. What is it? Why women get it?
Kiki? Lwaaki abakyala babufuna?

b. What type (who) of women are susceptible to GDM?
Kika ki ekyabakyala ekyangu okufuna obulwadde bwa sukaali?

c. How is GDM diagnosed/tested?
Obulwadde bwa sukaali mu bakyala babukebera batya?

d. What is the available treatment for GDM?
Obulwadde bwa sukali bukosa butya abakyala b'embuto?

e. How GDM affect pregnant women?
Obulwadde bwa sukali bukosa butya abakyala b'embuto?

f. How one should take care of GDM?
Omulwadde wa sukaali alina okwelabilira atya?

21. What exactly did your doctor tell you about GDM?
Omusawo wo yakugamba ki ku bulwadde bwa sukaali?

a. Messages/information
Obubaka/amawulire

b. Treatments suggestions
Obujjanjabi obutesedwaako

c. Lifestyle changes etc
Eneyisa oba obulamu obwa bulijjo

22. Other than the doctor, who else / where else do women go for additional health information (e.g. GDM)? And, Why?
Nga ogyeko omusawo, wa awalala wofuna ebisingako ku bikwata ku byobulamu? Nga obulwadde bwa sukaali?

a. Did you all seek additional information regarding GDM? Where? And why?
Wanoonya amawulire agasingako ku bulwadde bwa sukali?Waganonya wa? Ate lwaaki?

23. Whom did you reveal about your GDM? Why? And how did you reveal?
Ani gwe wabulirako ku bulwadde bwa sukaali? Lwaaki? Wamubulira otya?

a. If not revealed, why not?
Bwoba tolina gwe wabulirako, lwaaki?

24. Would you like to know more about GDM?
Kiki ekisinga ko kyoyagala okumanya?

a. Why?
Lwaki ?

b. What more do you all like to know?
Kiki ekirala kye wandyagadde okumanya ?

c. How would you like to receive the information?
Ngeri ki gy'oyagala okufunamu amawulire wa mmanga?

i. Poster /printed material
Poster/ kipande

ii. Film
Firimu

iii. Video etc.
Akatambi

iv. In what language?
Mu lulimi ki

26. What are your concerns related to GDM? Please tell us.
Tusaba otubulire biki ebikweralikiriza ku nsonga y'obulwadde bwa sukaali?

Experience of living with GDM:

27. Please tell us about your experience of knowing/learning about your GDM status?
Tubulireko bye wayitamu oluvanyuma lw'okukizuula nti olina obulwadde bwa sukali?

a. Who told you that you have GDM?
Ani eyakubulira nti olina obulwadde bwa sukaali?

b. What did she/he exactly tell?
Kiki kyenyini kyeyakugamba?

c. How did she/he explain?
Kiki kyenyini kyeyakugamba? Yakunyonyola atya?

d. Did you understand?
Byeyakunyonyola wa bitegeera?

28. How did you all feel when you first learned that you have been diagnosed with GDM?
Wawulira otya bwe wamanya nti olina obulwadde bwa sukali?

29. What has been the response of your family towards you having GDM?
Ab'enganda zo bayisibwa batya/bakitwala batya kukyo kuba omulwadde wa sukaali

a. How did they react? What did they say? Who said what!
Beyiisa batya?Bagamba batya? Ate biki bye bayogera

30. How GDM affected life?
    Obulwadde bwa sukali bukoseza butya obulamu bwo?

    a. What changes that women need to make in order to manage GDM? Did you all make those changes!
       Nkyukyuka ki abakyala ze betaaga okulaba nga bajanjaba obulwadde bwa sukali? Mwena mwakola enkyukakyuka ezo?

    b. How GDM affects pregnancy?
       Obulwadde bukosa butya omukyala ali olubuto?

31. How do you manage (monitoring sugar level) GDM? Who helps? and how
    Obulwadde bwa sukali obwekeneenya/obulondoola otya? Ani akuyamba? Akuyamba atya?

32. What are the difficulties in managing GDM (difficulties to take care regarding GDM)?
    Bizibu ki bwo sanga mu kwejanjaba obulwadde bwa sukali?

33. What kind of support do women need to take a better care to manage GDM?
    Buyambi ki abakyala abalina obulwadde bwa sukaali bwe betaaga okubera wo nga tebayisidwa bubi?

    a. What kind of support you think should GDM women receive from the families?

       Buyambi ki abakyala abalina obulwadde bwa sukali bwe balina okufuna okuva eri ab'enganda zaabwe?

Conclude the session thanking the participants. Stay there for about 10 minutes to ensure that the participant is comfortable, and get back to their social world (work). If respondents need any health care service, guide them to seek assistance from relevant healthcare providers

Thank you for your participation and co-operation. Have a good day!

In-depth Interview (IDI) Guide: Health Care Provider- Doctor

INTERVIEW INFORMATION:

Interviewer code	
Interview venue	
Interview date	
Interview start time	
Interview end time	
Total recorded time (hh/mm/)	

RESPONDENT INFORMATION:

Respondent type	Doctor
Hospital Code	
Designation	
Gender	
Age	
Education/qualification	
Experience (yy/mm)	

OBSERVATION:

- Look for IEC displayed in the waiting area (near the antenatal clinic).
- Look if there is anything specific on GDM (note/take pictures with permission from the hospital)

GUIDE:

Provider's Perspectives about pregnant women, screening, and GDM:

1. What is the profile (age, socio-economic and educational, language) of women who visit this hospital for pregnancy care?
2. Please tell us about the screening pregnant women?
    a. What are some of the screening tests done?
    b. When (at what month of pregnancy) are they done? Explain the process
3. Now, we would like to know specifically about GDM screening.
    a. In your hospital, are pregnant women screened for GDM?
    b. How common is GDM among women who visit your hospital?
        i. How many out of every 10 cases do you find women with GDM?
    c. What type of women are more susceptible to GDM (age, ethnicity, region/place, education, socio-economic status)?
4. How important is it to screen women for GDM? Why?
    a. How does screening help you (doctor)?
    b. How does it help women and her family?

5. What are the challenges to screen women for GDM? How to overcome such challenges?

6. What are the gaps in the current screening processes/protocols?

7. What are your suggestions to improve current screening process?

Processes and role in GDM screening:

8. Please explain the screening process- How does it start?
    a. Who: Who (hospital staff) are involved in screening?
    b. When: Is there any fixed day/ time for GDM screening? Why?
    c. How: how many tests do women need to undergo to know her GDM status?
    d. How do you prepare women for GDM screening?
        i. How do you plan screening activities?
        i. What are the conditions (if any) for the women to undergo GDM screening?
        ii. Who tells them about the rules/ conditions? When are they told about them?
    e. How much do you charge a woman to screen for GDM?

Post-screening process:

9. What happens after screening?
    a. How many days does it take for women to get the test results?
    b. Who discloses the GDM result to women? Do you?
        i. What exactly do you tell women with GDM? How do you explain?
        ii. What exactly do you tell women with **no** GDM?

Role and responsibilities in GDM screening:

10. What specific role do you play in screening?
    a. During screening/Diagnosis: what do you do?

b. Post-screening: what do you do (e.g. GDM education/ messaging/ empathizing etc)?

i. Do you provide any information/messages to women about GDM?
ii. Why/why not?
iii. What messages do you provide?

11. How does screening women for GDM help a doctor?
12. What challenges do you face in screening women for GDM?
13. What are the feelings/emotions/concerns of women and her family expressed after learning the GDM status?

Processes and role in GDM Treatment and Management:

14.What treatment women with GDM receive?
15. Who guide women about the treatment procedures/ management of GDM?
16. What role do you play in the GDM management (check sugar levels)?
17. What challenges do women face in the management of GDM? How does that affect you?

GDM Knowledge: Guidelines, Prevention and Treatment

18. What are the National guidelines for screening women for GDM?
19. Please tell me about GDM. What do you know about GDM?
    a. What causes GDM?
    b. How to prevent it?
20. What are the available treatment options for those diagnosed with GDM?
    a. Treatment types/criteria
    b. Medications
    c. Monitoring (of glucose)
21. When (at what stage of pregnancy) should women be diagnosed for GDM?
    a. What happens GDM diagnosis is delayed?
    b. What are the reasons/possibilities for delay?

Knowledge about GDM consequences:

22. How does GDM affect women?

a. During pregnancy
b. At the time of delivery
c. Post-delivery
d. How does it affect the baby?

GDM Resources: Infrastructure, Manpower and Skill sets:

23. Did you receive any training related to GDM?
    a. What was the name of the training programme?
    b. What was the content of the training programme (e.g. guidelines, management of GDM etc.)?
    c. When did that happen? And, where?

d. How many days were you trained? How was it in terms of learning about GDM

24. Would you require further training? Why? Why not?

25. What support (materials/otherwise) would you require for the effective management of GDM?

26. What are some of the IEC material that you use/give away during antenatal check-up? (collect/ see the material/ take picture)

25. Do you have any IEC materials specific to GDM? (collect if any)
   a. Would you require? Why/why not
   b. According to you what kind of IEC materials (posters, flyers or video/ film etc. would be helpful for pregnant women? Why?

27.What are the most important resources requirered for the effective screening and management of the GDM?

   a. man power (nurse/staff)
   b. infrastructure- laboratory, privacy etc.
   c. supplies - test kits

Conclude the session thanking the participant. Stay there for about 10 minutes to ensure that the participant is comfortable, and get back to their social world (work).

Thank you for your participation and co-operation. Have a good day!

In-depth Interview (IDI) Guide: Family Members of women with GDM

INTERVIEW INFORMATION:

Interviewer code	
Interview venue	
Interview date	
Interview start time	
Interview end time	
Total recorded time (hh/mm/)	

RESPONDENT INFORMATION:

Respondent type	Family member
Relationship with the pregnant woman	
Gender	
Age	
Education	
Employment status	
Economic status (monthly family income)	
Religion	
Family Type (Joint/ Nuclear)	
Total no of family members	
No of Diabetes patients within Family	

OBSERVATION:

GUIDE:

Pregnancy: Relevance and Perception:

1. What does it mean to be healthy?
   Kitegeza ki okuba omulamu?

2. How valuable is women's health in our society?
Obulamu bw'omukyala bwamuwendo butya mukitundu kyaffe?

   c. What factors influence women to take care of health?
   Biki ebiretera abakyala okulabirira obulamu bwabwe?

   d. What stops women from taking care of health?
   Kiki ekiziyiza omukyala okulabirira obulamu bwe?

Family: Dynamics, and pregnancy care decisions within the family

3. Generally, who in the family guides/advice women about pregnancy care?
   Okutwaliza awamu ,ani mu maka awa omukyala amagezi ku ndabirira y'olubuto

   c. How do you support pregnant woman?
   Oyamba otya omukyala ali olubuto?

   d. What do women do in absence of family support?
   Abakyala bakola batya awatali buyambi bwa famire?

4. Tell me about the decision-making process (with respect to health care seeking) within your family.
   Mbulira ku mitendera gye muyitamu okusalawo, (nga byekuusa ku kunoonya obujanjabi ewaka?)

   a. Who in the family makes decisions (which hospital/doctor, when to visit, etc) regarding health care seeking?
Ani mu makaago/gamwe asalawo ku by'obulamu (ddwaliro/musawoki, ddi lwe mugenda mudwaliro ne birala)?

   b. How those decisions are made?
   Okusalawo kukolebwa kutya?

   c. On what basis (criteria- e.g. money, distance, parityetc) decisions are made?
   Okusalawo kuno kukolebwa kutya? (emitendera-nga Sente, obuwanvu, omwenkanokano nebirala)

   d. Tell me about women's involvement in decision making with respect to;
   Mbulira okwenyigira kwo mukusalawo ku bino wamanga;

iii. Getting pregnant/ having children
Ebyo kufuna olubuto / okuzaala abaana

iv. Choosing a hospital, and the doctor
Ekusalawo eddwaliro wamu n'omusawo

a. What are those problems (tensions) within your family that affect the pregnancy care? And how? (Probe for Finance, Time, any other?)
Bizibuki/Mitawaana ki mu maka gamwe ebikosa endabirira y'olubuto? Ate bitya? (Mubyenfuna, obudde, nebirirala)

Culture: pregnancy-related beliefs and practices:

5. What are the beliefs and rituals followed during pregnancy? How do they help?
Nzikiriza ki n'obulombolombo abantu bwe bagoberera nga bali mbuto? Biyamba bitya?

c. Are these ceremonies/rituals specific to your tribe/community?
Gino emikolo/obulombolombo bukozesebwa mu kitundu kyo ky'obelamu?

6. What are the expected (behaviors) of a pregnant woman? What are your expectations? (Probe for Going to hospital, Taking injections/tablets, Doing routine work, Rest /sleep Food, Cloths)
Omukyala w'olubuto alina kweyisa atya?(Ngangenda mudwaliro, Nga funa empiso/ edagala, Nga akola emirimu egyabulijjo, Ng'awumudemu/yebase, Mubyendya, Munyambala

7. Please tell about the where you live (which locality)?
Nsaba ombulire gy'obeera? (kitunduki)

d. What type of population groups (by religion, economic class, education, occupation etc) live there?
Bantu kika ki (mubyenzikiriza, ebyenfuna, mubyenjigiriza, n'emirimu gyebakola) ababeera mukitundu gy'obeera

e. How do neighbors help you? Do you discuss pregnancy related information with your neighbours?
Baliranwa bo bakuyamba batya? Otera okunyumyamu nebalirwanabo ku bikwata ku by'okubera olubuto?

f. How well connected to seek health care? What are benefits living in this area?
Kikwanguyira kitya okufuna obujjanjabi?Birungi ki ebiri mu kubeera mu kitundu kino?

Learning about GDM:

8. Please tell us about your experience of knowing/learning about your wife's/daughter-in-law's GDM status?
   Tubulire ku by'oyiseemu mu kumanya / mukusoma ku bulwadde bwa sukali obwa mukyaala/ muka mwana wo?

   e. Who told you about it?
      Ani ayakubulira ku bulwadde bunno?

   f. What did she/he exactly tell?
      Kiki kyenyini kyeyakugamba?

   g. How did she/he explain?
   Yakunyonyola atya?

   h. Did you understand?
      Byeyakunyonyola wa bitegeera?

9. How did you all feel when you first learned about your wife's/daughter-in-law's GDM status?
   Wawulira otya bwe wamanya nti omukyala/ muka mwana alina obulwadde bwa sukaali?

10. How did your wife/daughter-in-lawrespond? What did they say? Who said what!
    Mukyala wo/mukamwana wo yayisibwa atya,? **Bagamba batya? Ate biki byebayogera?**

11. How GDM affected her?
    Obulwadde bwa sukaali bukoseza butya obulamu bwe?

c. What changes she made in order to manage GDM?
Nkyukyuka ki z'akoze okulaba ng'ajanjaba obulwadde bwa sukaali?

d. How do you support her in managing GDM? Why?
Omuyamba otya okujanjaba obulwadde bwa sukaali ?

Knowledge specific to GDM:

Generally, some women are susceptible to a few health complications during pregnancy. GDM is one such condition, where blood sugar level increases. Now, we would like to know more from you, about GDM.

Okutwaliza wamu, abakyala abamu bali mukatyabaga k'okufuna obuzibu nga bali mbuto. Obulwadde bwa sukali bwe bumu ku bwo. Twandyagadde okumanya ekisingako okuva gy'oli ebikwata ku sukali

12. What is GDM? Why women get it?
    Obulwade bwa sukali butegeza ki?Lwaaki abakyaala ba bufuna?

    g. What type (who) of women are susceptible to GDM?
    Kika ki ekyabakyala ekyangu okufuna obulwadde bwa sukaali?

    h. How is GDM diagnosed/tested?
    Obulwadde bwa sukaali mu bakyala babukebera batya?

b. What is the available treatment for GDM?
   Sukali alina bujanjabi ki?

    a. How GDM affect pregnant women?
    Obulwadde bwa sukali bukosa butya abakyala b'embuto?

    b. How one should take care of GDM?
    Omulwadde wa sukaali alina okwerabirira atya?

13. What exactly did your doctor tell you about GDM?
    Omusawo wo yakugamba/ oba yagamba ki mukyala wo/mukamwana wo ku bulwadde bwa sukaali?

    d. Messages/information
    Obubaka/amawulire

    e. Treatments suggestions
    Obujjanjabi obutesedwaako

    f. Lifestyle changes etc
    Eneyisa oba obulamu obwa bulijjo

14. Other than the doctor, who else / where else do you seek additional health information (e.g. GDM)? And, Why?
    Nga ogyeko omusawo, walala wa wo funa ebisingako kubikwata ku byobulamu? Nga obulwadde bwa sukaali

    b. Did you all seek additional information regarding GDM? Where? And why?
    Wanoonya amawulire agasingako ku bulwadde bwa sukali?Waganonya wa? Ate lwaaki?

15. Whom did you reveal about your (wife/ daughter- in -law's) GDM status? Why? If not revealed, why not?
    Ani gwe wabulirako ku bulwadde bwa sukaali?Lwaaki?Wamubulira otya?
    Bwoba tolina gwe wabulirako lwaaki?

16. Would you like to know more about GDM?
    Oyagala okumanya ebisingako ku bulwadde bwa sukali?

d. Why?

Lwaaki?

e. What more do you all like to know?

Kiki ekisingako kyoyagala okumanya?

f. How would you like to receive the information?

Ngeri ki gy'oyagala okufunamu amawulire wa mmanga?

i. Poster /printed material

Poster/ kipande

ii. Film

Firimu

iii. Video etc.

Akatambi

iv. In what language?

Mu lulimi ki

17. What are your concerns related to GDM? Please tell us.
Tusaba otubulire biki ebikweralikiriza ku nsonga y'obulwadde bwa sukali?

Consequence of GDM on family:

18. How your wife/ daughter-in-law having GDM has affected the family?
Omukyala wo oba mukamwana wo okubeera n'oblwadde bwa sukali, kikoseza kitya amaka gamwe?

a. The health of the pregnant (currently) woman

Obulamu bw'omukyala ali lubuto

b. Financial, Emotional

Ebyenfuna

c. What kind of support would you require to deal with your wife/ daughter-in-law's GDM status?
Buyambi ki obwetagisa okulabirira omukyala wo/Mukamwana wo ng'alina obulwadde bwa sukaali?

i. From whom? How?
Okuva wa? Otya?

Experience of seeking health (Pregnancy) care services:

19 Tell me about your experience of seeking pregnancy care in this hospital (mention the name)?

Mbulira by'oyiseemu mukunywa eddagala nga oli lubuto mudwaliro lino (Yogera erinya ly'eddwaliro).

d. How is the doctor? How does he/she treat your wife/ daughter- in -law?
Omusawo ali atya? Era akuyisa atya?

e. Does he/she give adequate information/ and reasonable time etc
Akuwa amawulire agasaana n'obudde obumala?

f. How other staff (nurse/ lab technician) treat?
Abasawo abalala bakuyisa batya?

20. What do you like about this hospital?
Kiki kye wenyumirizamu kudwaliro lino?

21. What do you NOT like about this hospital?
Kiki kyotayagala ku dwaliro lino?

22. What are suggestions to improve the patient experience at this hospital?
Biki by'olowooza ebya ndikoleddwa ku mutindo gw'obujanjabi mu dwaliro lino?

Conclude the interview thanking the participant. Stay there for about 10 minutes to ensure that the respondent is comfortable, and get back to their social world (work).

Thank you for your participation and co-operation. Have a good day!

In-depth Interview (IDI) Guide: Women with GDM

Hospital Code:

INTERVIEW INFORMATION:

Interviewer/moderator code	
Venue	
Interview date	
Start time	
End time	
Total recorded time (hh/mm)	

RESPONDENT (R) INFORMATION:

Respondent code	PegWGDM No
Age	
Religion	
Education (self)	
Education (spouse)	
Employment status	
Family Type (joint/nuclear)	
Economic status (monthly family income)	
Language (mother tongue)	
No of children	
Pregnancy order	
Pregnancy Trimester	

Diabetes history (family)	
Diabetes History (self)	

OBSERVATION:

Pregnancy: Relevance, Knowledge and Experience

1. What does it mean to be healthy?
Kitegeeza ki okuba omulamu ?

2. How valuable is women's health in our society?
Obulamu bw'omukyala bwamuwendo butya mukitundu kyaffe?

e. What factors make women to take care of health?
Biki ebiretera abakyala okulabirira obulamu bwaabwe?

f. What stops her from taking care of health?
Kiki ekiziyiza omukyala okulabilila obulamu bwe?

3. How important is pregnancy for a woman?
Okuba olubuto kya mugaso ki eri omukyala /olubuto lugasa lutya omukyala?

b. What changes it brings in life:
Enkyukakyuka olubuto lwezireta mu bulamu

Positive changes due to pregnancy
Enkyukakyuka ennungi mukubeera olubuto

iii. Difficulties of being pregnant.
Obuzibu mu kubeera olubuto

4. Please tell me about your current pregnancy?
Nsaba ombulire ku bikwata ku lubuto lw'olina kati?

a. How is your pregnancy going on?
Olubuto lukuyisa lutya?

b. What specific care do you take to ensure that you stay well during this time (pregnancy)?
Biki by'okola okulaba nti osigala ng'oli mulamu mu kaseera kano ng'oli lubuto.

5. How do you know if a pregnancy is going well (if everything is fine)?
Omanya otya nti olubuto lwo luli bulungi? (nti buli kimu kiri bulungi)

b. What are those signs/symptoms that indicate you that you are doing well?

Bubonero ki obulaga nti oli bulungi?

c. How did you learn these things?
Ebintu bino wabiyiga otya?

d. Did you have any such symptoms? What did you do to overcome them?
Walina ko obubonero bwe butyo?Wabuvunuka otya?

6. Who guides/advises you about pregnancy care: what to do/ what not to do?

Ani akuwa amagezi ku by'okulabirira olubuto?

a. Whose advice do you value the most? Why?
   Magezi g'ani gosinga okutwala? Iwaki?

Family: Dynamics and pregnancy care decisions within the family

7. Now, tell me who within family supports you during pregnancy?
   Mbulira ewaka gy'obeera ani asinga okukuyamba ng'oli lubuto?

   a. How?
      Akuyamba atya?

   b. What care/attention are you receiving?

      Buyambi ki bwofuna?

8. How does your husband support you during this time (pregnancy)?
   Omwami wo akuyamba atya ng'oli lubuto?

   a. How pregnancy has affected your relationship with your husband?

   Enkolagana yo n'omwami wo ebadde etya ng'oli lubuto luno?

      i. Positive changes in your relationship due to pregnancy
      Enkyukakyuka ennungi ezireteddwa munkolagana wakati wo nomwami wo nga ziva kukubeera n'olubuto

      ii. Disturbance in the relationship due to pregnancy
      Obutategeeragana mu mukwano gwamwe nga buva kukubeera n'olubuto.

9. What are those problems (tensions) within your family that affect the pregnancy care? And how? (Probe for Financial, Behavioural- violence, alcoholic members, Social- preference for son, Relationships dynamics: In-laws, Husband- wife power etc)
   Bizibuki/Mitawaana ki mu maka gammwe ebikosa endabirira y'olubuto? Bikosa bitya? (Mubyenfuna, Muneyisa, okulwana, abanywi b'omwenge awaka, Okwagala enyo omwana ow'obulenzi, Enkyukakyuka mu mukwano: Ab'enganda, obuyinza wakati wo n'omwami wo)

10. Tell me about the decision-making process (with respect to health care seeking) within a family.

Mbulira ku mitendera gye muyitamu okusalawo, (nga byekuusa ku kunoonya obujanjabi ewaka?)

a. Who in your family makes decisions (which hospital/doctor, when to visit, etc) regarding health care? How those decisions are made?

Ani mu makaago/gammwe asalawo ku by'obulamu (ddwaliroki/musawoki, ddi lwe mugenda muddwaliro ne birala)

b. On what basis (criteria- e.g. money, distance, parity etc) decisions are made?
Okusalawo kuno kukolebwa kutya? (emitendera-nga Sente, olugendo, omwenkanokano nebirala)

c. Tell me about your involvement in decision making with respect to;
Mbulira okwenyigira kwo mukusalawo ku bino wammanga;

v. Getting pregnant/ having children
Ebyo kufuna olubuto / okuzaala abaana

vi. Choosing a hospital, and the doctor
Ekusalawo eddwaliro wamu n'omusawo

vii. Who made decisions about getting pregnant (with respect to current one)?
Ani yasalawo okufuna olubuto naddala luno lw'olina kati

viii. Who decided to seek healthcare from the hospital from where you are currently seeking health care?
Ani yasalawo okugenda muddwaliro gy'ofunira obujjanjabi kati?

Culture: pregnancy-related beliefs and practices:

11. What are the beliefs and rituals followed during pregnancy? How do they help?

Nzikiriza ki n'obulombolombo abantu bwe bagoberera nga bali mbuto? Biyamba bitya?

a. Please tell me about the ceremonies/rituals you practice/follow. Why did you do that?
Nsaba ombulire emikolo/obulombolombo bwe wakozesa? Era lwaki wa bukozesa?

12. Are these ceremonies/rituals specific to your caste/community?
Gino emikolo/obulombolombo businga bukozesebwa mu kitundu kyo ky'obelamu?

13. What beliefs and practices make the life of pregnant women difficult? How?
Nzikiriza ki awamu n'obulombolombo obukalubiliza obulamu bw'abakyala abe mbuto?

14. What are the expected (behaviors)of a pregnant woman? (Probe for Going to hospital, Taking injections/tablets, Doing routine work, Rest /sleep, Food, Cloths)

Omukyala w'olubuto alina kweyisa atya?(Ng'agenda muddwaliro, ng'afuna empiso/ eddagala, ng'akola emilimu egyabulijjo, ng'awumudemu/yeebase, Mubyendya, Munyambala

15. Please tell me about the where you live (which locality)?
    Nsaba ombulire gy'obeera? (kitunduki)

    g. What type of population groups (by religion, economic class, education, occupation etc) live there?
       Bantu kika ki (mubyenzikiriza, ebyenfuna, mubyenjigiliza, n'emirimu gyebakola) ababeera mukitundu gy'obeera
    h. How do neighbors help you? Do you discuss pregnancy related information with your neighbours
       Balirwanabo bakuyamba batya? Otera okunyumyaamu nebalirwanabo ku bikwata ku by'okubera olubuto?

    i. How well connected are you to seek health care? What are the benefits of living in this area?
       Kikwanguyira kitya okufuna obujjanjabi?Birungi ki ebiri mu kubeera mu kitundu kino?

Health Information seeking:

14. Where do you seek information about health care, particularly pregnancy care?From whom?From where? Or How? (Probe for magazine, internet, social media, WhatsApp/Facebook, TV/Radio etc, Friends/ family/relatives, Neighbors)
Ofunirawa amawulire agakwata ku by'obujanjabi okusingila dala ku by'okulabilira olubuto? Ani akuwa amawulire ago? Okuva wa? Amawulire ogafuna otya? (Empapula/obutabo, Omutimbagano, WhatsApp/ Facebook, Telefayina/ radiyo, Emikwaano/amaka/abenganda, Balirwana)

Experience of seeking health (pregnancy) care services:

16. Tell me about your experience of seeking pregnancy care in this hospital (mention the name)?
    Mbulira byoyiseemu mukunywa eddagala ng'oli lubuto muddwaliro lino (Yogera erinya ly'eddwaliro).

    g. How is the doctor? How does he/she treat you?
       Omusawo ali atya? Era akuyisa atya?

    h. Does he/she give adequate information/ and reasonable time etc
       Akuwa amawulire agasaana n'obudde obumala?

    i. How do other staffs (nurse/ lab technician) treat you?
       Abasawo abalala bakuyisa batya?

17. What do you like about this hospital/health centre?
    Kiki kyewenyumirizamu kuddwaliro lino?

18. What do you NOT like about this hospital/health centre?
    Kiki kyotayagala ku ddwaliro lino?

19. What are suggestions to improve the patient experience at this hospital/health centre?

Biki byolowooza ebyandikoleddwa ku mutindo gw'obujjanjabi mu ddwaliro lino?

Knowledge specific to GDM:

Generally, some women are susceptible to a few health complications during pregnancy. GDM is one such condition, where blood sugar level increases. Now, we would like to know more from you, about GDM.
Okutwaliz'awamu, abakyala abamu bali mukatyabaga kokufuna obuzibu nga bali mbuto. Obulwadde bwa sukali bwe bumu ku bwo. Twandyagadde okumanya ekisingako okuva gy'oli ebikwata ku sukali

20. What is GDM? And why women get it?
    Obulwade bwa sukali obutegeera otya?Lwaaki abakyaala ba bufuna?

    c. What type (who) of women are susceptible to GDM?

    Kika ki ekyabakyala ekyangu okufuna obulwadde bwa sukaali?

    d. How is GDM diagnosed/tested?

    Obulwadde bwa sukaali mu bakyala babukebera batya?

    e. What is the available treatment for GDM?

    Sukali ajjanjabibwa atya?

    f. How does GDM affect pregnant women?
    Obulwadde bwa sukali bukosa butya abakyala b'embuto?

    g. How should one take care of GDM?

    Omulwadde wa sukaali alina kwelabilira atya?

21. What exactly did your doctor tell you about GDM?
    Omusawo wo yakugamba ki ku bulwadde bwa sukaali?

    g. Messages/information
    Bubaka ki/amawulire ki geyakuwa?

    h. Treatments suggestions
    Bujjanjabi ki bwemwatesaako

    i. Lifestyle changes etc
    Nkyukakyukaki zemwatesaako mu nneyisa oba mubulamu obwa bulijjo

21. Other than the doctor, where else do you seek additional health information (e.g. GDM)?
Ng'gyeko omusawo, bifo ki ebirala gyofuna ebisingako kubikwata ku by'obulamu, ng'obulwadde bwa sukaali?

c. Did you seek additional information regarding GDM? Where? And why?
Wanoonya amawulire agasinga ko ku bulwadde bwa sukali?Waganonya wa? Ate lwaaki?

22. Whom did you reveal to about your GDM? Why? And how did you reveal?
Ani gwe wabulirako ku bulwadde bwa sukaali?Lwaaki?Wamubulira otya?

b. If not revealed, why not?
Bwoba tolina gwe wabulirako, lwaaki kiri bwekityo?

23. Would you like to know more about GDM?
Oyagala okumanya ebisingawo ku bulwadde bwa sukaali?

g. Why?
Lwaaki?

h. What more do you or like to know?
Kiki ekirala kyoyagala okumanya?

i. How would you like to receive the information?
Ngeri ki gy'oyagala okufunamu amawulire gano wammanga?

i. Poster /printed material
Poster/ kipande
ii. Film
Filimu
iii. Video etc.
Akatambi
iv. In what language
Mu lulimi ki

24. What are your concerns related to GDM? Please tell us.
Tusaba otubulire biki ebikweralikiriza ku nsonga y'obulwadde bwa sukaali?

Experience of living with GDM:

25. Please tell us about your experience of knowing/learning about your GDM status?
Tubulireko bye wayitamu oluvanyuma lw'okukizuula nti olina obulwadde bwa sukaali?

i. Who told you that you have GDM? Probes (What did she/he exactly tell?, How did she/he explain?

Ani eyakubulira nti olina obulwadde bwa sukaali? (Kiki kyenyini kyeyakugamba? Yakunyonyola atya?)

j. Did you understand?
Byeyakunyonyola wa bitegeera?

25. Please tell us about how you felt when you were diagnosed with GDM?
Wawulira otya bwe wamanya nti olina obulwadde bwa sukaali?

27. What has been the response of your family towards you having GDM?
Ab'enganda zo bayisibwa batya/bakitwala batya kukyokuba omulwadde wa sukaali

a. How did they react? What did they say? Who said what!
k. **Beyiisa batya?Bagamba batya? Ate biki bye bayogera**

26. How GDM affected life?
Obulwadde bwa sukaali bukoseza butya obulamu bwo?

e. What changes do women need to make in order to manage GDM? Did you make those changes!
Nkyukyuka ki abakyala ze betaaga okulaba nga bajanjaba obulwadde bwa sukaali? Wassa munkola enkyukakyuka ezo?
f. How does GDM affect pregnancy?
Obulwadde bukosa butya omukyala ali olubuto?

27. How do you manage (monitoring sugar level) GDM? Who helps? and how?
Obulwadde bwa suknali obwekeneenya/obulondoola otya? Ani akuyamba? Akuyamba atya?

28. What are the difficulties in managing GDM (difficulties to take care regarding GDM)
Bizibu ki bwosanga mu kwejanjaba obulwadde bwa sukaali?

29. What kind of support do women need to manage GDM?
Buyambi ki abakyala abalina obulwadde bwa sukaali bwebetaaga okuberawo nga tebayisiddwa bubi?

b. What kind of support do you think womenwith GDM should receive from the families?
Buyambi ki abakyala abalina obulwadde bwa sukali bwe balina okufuna okuva eri ab'enganda zaabwe?

Conclude the interview thanking the participant. Stay there for about 10 minutes to ensure that the respondent is comfortable, and get back to their social world (work).

Thank you for your participation and co-operation. Have a good day!

KEY INFORMANT INTERVIEWS (KII) Guide: Health Care Provider- NURSE

INTERVIEW INFORMATION:

Interviewer code	
Interview venue	
Interview date	
Interview start time	
Interview end time	
Total recorded time (hh/mm/)	

RESPONDENT INFORMATION:

Respondent type	Nurse
Hospital Code	
Designation	
Gender	
Age	
Education/qualification	
Experience (yy/mm)	

OBSERVATION:

- Look for IEC displayed in the waiting area (near the antenatal clinic).
- Look if there is anything specific on GDM (note/take pictures with permission from the hospital)

GUIDE:

Provider's Perspectives about pregnant women, screening, and GDM:

1. What is the profile (age, socio-economic and educational, language) of women who visit this hospital for pregnancy care?
2. Please tell us about the screening of pregnant women:
   c. What are some of the screening tests done?
   d. When (at what month of pregnancy) are they done? Explain the process

[if GDM is mentioned, first obtain the details of all the other screening tests]

3. Now, we would like to know specifically about GDM screening.
   d. In your hospital, are pregnant women screened for GDM? [if no, ask Qn 16}
   e. How common is GDM among women who visit your hospital?
      i. How many out of every 10 cases do you find women with GDM?
   f. What type of women are more susceptible to GDM (age, ethnicity, region/place, education, socio-economic status)?
4. How important is it to screen women for GDM? Why?
   c. How does it help women and her family?

Processes and role in GDM screening:

5. Please explain the screening process- How does it start?
   f. Who: Who (hospital staff) are involved in screening?
   g. When: Is there any fixed day/ time for GDM screening? Why?
   h. How: how many tests do women need to undergo to know her GDM status?
   i. How do you prepare women for GDM screening?
      i. How do you plan screening activities?
      iii. What are the conditions (if any) for the women to undergo GDM screening?
   j. How much do you charge a woman to screen for GDM?

Post-screening process:

6. What happens after screening?
   c. How many days does it take for women to get the test results?
   d. Who discloses the GDM result to women?
      i. What is told to women with GDM?
      ii. What is told to women with no GDM?

Role and responsibilities in GDM screening:

7. What specific role do you play in screening?
   a. During screening/Diagnosis: what do you do?
   b. Post-screening: what do you do (e.g. GDM education/ messaging/ empathizing etc)?
      iv. Do you provide any information/messages to women about GDM?
      v. Why/why not?
      vi. What messages do you provide?
8. How does screening women for GDM help a nurse?
9. What challenges do you face in screening women for GDM?

10. What are the feelings/emotions/concerns of women and her family expressed after learning the GDM status?

Processes and role in GDM Treatment and Management:

11. What treatment women with GDM receive?
12. Who guide women about the treatment procedures/ management of GDM?
13. What role do you play in the GDM management (check sugar levels)?
14. What challenges do you face in supporting women manage GDM?
15. What challenges do women face in the management of GDM? How does that affect you?

GDM Knowledge: Guidelines, Prevention and Treatment

16. What are the National guidelines for screening women for GDM?
17. Please tell me about GDM. What do you know about GDM?
    c. What causes GDM?
    d. How to prevent it?
18. What are the available treatment options for those diagnosed with GDM?
    d. Treatment types/criteria
    e. Medications
    f. Monitoring (of glucose)
19. When (at what stage of pregnancy) should women be diagnosed for GDM?
    c. What happens GDM diagnosis is delayed?
    d. What are the reasons/possibilities for delay?

Knowledge about GDM consequences:

20. How does GDM affect women?
    a. During pregnancy
    b. At the time of delivery
    c. Post-delivery
    d. How does it affect the baby?

GDM Resources:

21. Did you receive any training related to GDM?
    a. What was the name of the training programme?
    b. What was the content of the training programme (e.g. guidelines, management of GDM etc.)?
    c. When did that happen? And, where?
    d. How many days were you trained? How was it in terms of learning about GDM
22. Would you require further training? Why? Why not?
23. What support (materials/otherwise) would you require for the effective management of GDM?
24. What are some of the IEC material that you use/give away during antenatal check-up? (collect/ see the material/ take picture)

25. Do you have any IEC materials specific to GDM? (collect if any)
    c. Would you require? Why/why not
    d. According to you what kind of IEC materials (posters, flyers or video/ film etc. would be helpful for pregnant women? Why?

Conclude the session thanking the participant. Stay there for about 10 minutes to ensure that the participant is comfortable, and get back to their social world (work).

Thank you for your participation and co-operation. Have a good day!
